# Data on Determinants Are Needed to Curb the Sedentary Epidemic in Europe. Lessons Learnt from the DEDIPAC European Knowledge Hub

**DOI:** 10.3390/ijerph15071406

**Published:** 2018-07-04

**Authors:** Marieke De Craemer, Sebastien Chastin, Wolfgang Ahrens, Claire Bernaards, Johannes Brug, Christoph Buck, Greet Cardon, Laura Capranica, Patricia Dargent-Molina, Sara De Lepeleere, Belinda Hoffmann, Aileen Kennedy, Jeroen Lakerveld, Nanna Lien, Fiona Ling, Anne Loyen, Ciaran MacDonncha, Julie-Anne Nazare, Grainne O’Donoghue, Donal O’Gorman, Camille Perchoux, Iris Pigeot, Chantal Simon, Annabel S. Mueller-Stierlin, Hidde van der Ploeg, Jelle Van Cauwenberg, Jean-Michel Oppert

**Affiliations:** 1 Department of Movement and Sports Sciences, Ghent University, 9000 Ghent, Belgium; sebastien.chastin@gcu.ac.uk (S.C.); greet.cardon@ugent.be (G.C.); sara.delepeleere@ugent.be (S.D.L.); 2 Institute for Applied Health Research, Glasgow Caledonian University, Glasgow G4 0BA, UK; 3 Leibniz Institute for Prevention Research and Epidemiology—BIPS, 28359 Bremen, Germany; Ahrens.dedipac@bips.uni-bremen.de (W.A.); buck@bips.uni-bremen.de (C.B.); pigeot@bips.uni-bremen.de (I.P.); 4 Amsterdam University of Applied Sciences, 1000 BA Amsterdam, The Netherlands; cbernaards@gmail.com; 5 Amsterdam Public Health Research Institute, VU University Medical Center, De Boelelaan 1089b, 1081 HV Amsterdam, The Netherlands; j.brug@vumc.nl (J.B.); j.lakerveld@vumc.nl (J.L.); anne.loyen88@gmail.com (A.L.); hp.vanderploeg@vumc.nl (H.v.d.P.); 6 University of Rome Foro Italico, 00135 Roma, Italy; laura.capranica@uniroma4.it; 7 Inserm UMR1153 Epidemiology and Biostatistics Sorbonne Paris Cité Center (CRESS), Early Determinants of Children’s Health and Development Team (ORCHAD), Villejuif 94807, France & Paris Descartes University, 75006 Paris, France; patricia.dargent@inserm.fr; 8 Division of Sports and Rehabilitation Medicine, Department of Medicine II, Ulm University, 89081 Ulm, Germany; Belinda.hoffmann@uni-ulm.de; 9 Centre for Preventive Medicine, Dublin City University, Dublin 9, Ireland; aileen.m.kennedy@dit.ie (A.K.); grainne.odonoghue@ucd.ie (G.O.); donal.ogorman@dcu.ie (D.O.); 10 Department of Nutrition, University of Oslo, 0316 Oslo, Norway; nanna.lien@medisin.uio.no; 11 Centre for Physical Activity and Health Research, University of Limerick, Limerick V94 T9PX, Ireland; Fiona.ling@ul.ie (F.L.); Ciaran.macdonncha@ul.ie (C.M.); 12 CARMEN, Inserm U1060, Université de Lyon 1, 69100 Villeurbanne, France; julie-anne.nazare@univ-lyon1.fr (J.-A.N.); chantal.simon@univ-lyon1.fr (C.S.); 13 Luxembourg Institute of Socio-Economic Research, Esch-sur-Alzette, 4366 Luxembourg, Luxembourg; Camille.perchoux@gmail.com; 14 Institute of Epidemiology and Medical Biometry, Ulm University, 89081 Ulm, Germany; Annabel.mueller-stierlin@uni-ulm.de; 15 Department of Public Health, Ghent University, 9000 Ghent, Belgium; jelle.vancauwenberg@ugent.be; 16 Department of Nutrition, University of Pierre et Marie Curie & Institute of Cardiometabolism and Nutrition (ICAN) & Pitie-Salpetrière Hospital (AP-HP), Paris 75013, France; jean-michel.oppert@psl.aphp.fr

**Keywords:** determinants, sedentary behaviour, European cohort, statement

## Abstract

Societal and technological changes have resulted in sitting being the dominant posture during most activities of daily living, such as learning, working, travelling and leisure time. Too much time spent in seated activities, referred to as sedentary behaviour, is a novel concern for public health as it is one of the key lifestyle causes of poor health. The European DEDIPAC (Determinants of Diet and Physical Activity) Knowledge Hub coordinated the work of 35 institutions across 12 European member states to investigate the determinants of sedentary behaviour. DEDIPAC reviewed current evidence, set a theoretical framework and harmonised the available epidemiological data. The main results are summarised. The conclusion is that there is a dire lack of data that is exploitable across Europe to inform policy and intervention. There is an urgent need to develop international data collection compliant with FAIR (Findable, Accessible, Interoperable, Re-usable) and standardised surveillance systems for sedentary behaviour.

FOREWORD

***International Endorsement for the DEDIPAC European Knowledge Hub by the Sedentary Behaviour Council of the International Society for Physical Activity and Health (ISPAH-SBC)***

 

**Andrew J. Atkin ^1^, Brigid M. Lynch ^2,3,4,5^, Katrien Wijndaele ^2,6^**

 

**on behalf of the Sedentary Behaviour Council of the International Society for Physical Activity and Health (ISPAH-SBC)**

^1^  School of Health Sciences, Faculty of Medicine and Health Sciences, University of East Anglia, Norwich, UK
^2^  Co-Chair, Sedentary Behaviour Council, International Society for Physical Activity and Health
^3^  Principal Fellow, Cancer Epidemiology and Intelligence Division, Cancer Council Victoria, Australia
^4^  Honorary Principal Fellow, Centre for Epidemiology and Biostatistics, Melbourne School of Population and Global Health, The University of Melbourne, Australia
^5^  Honorary Fellow, Physical Activity Laboratory, Baker Heart and Diabetes Institute, Australia
^6^  Senior Investigator Scientist, MRC Epidemiology Unit, University of Cambridge, UK

The field of sedentary behaviour research has expanded significantly in the last 30 years. From its early foundations in observational research exploring the associations of self- or proxy-reported television viewing with type 2 diabetes (1) and childhood obesity (2), the field has evolved to embrace a broad range of sedentary behaviour exposure and outcome variables, experimental as well as observational study designs, and a myriad of measurement tools. As a result, sedentary behaviour now sits alongside physical activity in many nations’ public health guidelines and attracts frequent attention within the mainstream media and policy discourse. Despite excellent progress, important gaps in our understanding remain. Guided by the behavioural epidemiology framework (3), one area in urgent need of further research lies in understanding the personal, social, political and environmental determinants of sedentary behaviour throughout the life course. Such information is essential to inform the design of effective behaviour change interventions, and to understand the optimal time for intervention. The paucity of research in this area limits opportunities to reduce sedentary behaviour prevalence and the associated burden of disease. Through a series of systematic literature reviews, and associated work packages, the DEDIPAC project has served to highlight the strengths, limitations, gaps and opportunities in sedentary behaviour surveillance and determinants research across Europe. This work is highly complementary to the Sedentary Behaviour Council’s mission of advancing science, advocacy and practice relating to sedentary behaviour and health. We wholeheartedly endorse the call for a sustained and widespread uplift in research exploring the determinants of sedentary behaviour, including the establishment of a pan-European cohort, as detailed by the DEDIPAC team in this special edition of IJERPH.

## 1. The Problem

Whether we move enough or not, is essential for our health. In parallel with the deleterious health effects of insufficient physical activity, too much sedentary behaviour (sitting) is recognized as a distinct risk factor for morbidity and mortality [1,2]. Nowadays, sedentary behaviour is ubiquitous; a hallmark of modern life affecting all European citizens, people living in middle- and high-income countries and a growing proportion of those living in low-income countries [3]. Societal changes have resulted in sitting being the dominant posture during most activities of daily living, such as learning, working, travelling and leisure time. The amount of time we spend sitting everyday has crept up almost unnoticed over the last 50 years, displacing many forms of physical activity and is forecasted to increase [3]. It is a silent epidemic carrying a high health and economic burden [4] that needs to be tackled.

To address this major societal challenge, the European member states initiated and facilitated the DEDIPAC (Determinants of Diet and Physical Activity) Knowledge Hub within the framework of the Joint Programming Initiative (JPI) “A Healthy Diet for a Healthy Life”, with a specific work programme focusing on sedentariness.

DEDIPAC regroups 68 consortia and research institutes from 13 EU Member States, and its major aim is to create a unified transdisciplinary vision among stakeholders to foster meaningful breakthroughs in the understanding of the determinants of sedentary behaviour necessary to the development of programmes, public health interventions and policies to reduce sedentary behaviour [5].

The objectives of the DEDIPAC working programme on sedentary behaviour are to (1) review the current evidence base; (2) develop a theoretical framework; and (3) use European diversity as a natural laboratory by creating an inventory of currently available data and methods.

## 2. Current Evidence Base on the Determinants of Sedentary Behaviour

A series of systematic literature reviews assessed the available evidence on factors influencing sedentary behaviour across the life course, in youth [6], adults [7] and older adults [8]. These revealed a disabling dearth of knowledge on the determinants of sedentary behaviour, particularly in older adults and youth. The few determinants and correlates identified generally say more about *who* is sedentary than *why* they are, giving information about whom interventions might be targeted towards but providing little scope for action. To date, the available studies have almost entirely focused on socio-demographic factors in isolation and neglected to investigate more distal contextual factors in the built, social and economic environment.

## 3. Framework

To foster more effective research and address these gaps, the DEDIPAC consortium established a system-based transdisciplinary framework called the SOS framework (Systems of Sedentary behaviours) [9] through an international consensus. The framework, for studying the determinants of sedentary behaviour and informing policy, maps 190 potential determinants identified across the life span and organizes them in a system of six interacting clusters: (1) Physical Health and Wellbeing; (2) Social and Cultural Context; (3) Built and Natural Environment; (4) Psychology and Behaviour; (5) Politics and Economics; and (6) Institutional and Home Settings. In addition, priorities were set to focus research on the potentially most modifiable and impactful parts of the system.

## 4. Existing Data Inventory and Analysis

**Figure 1:**
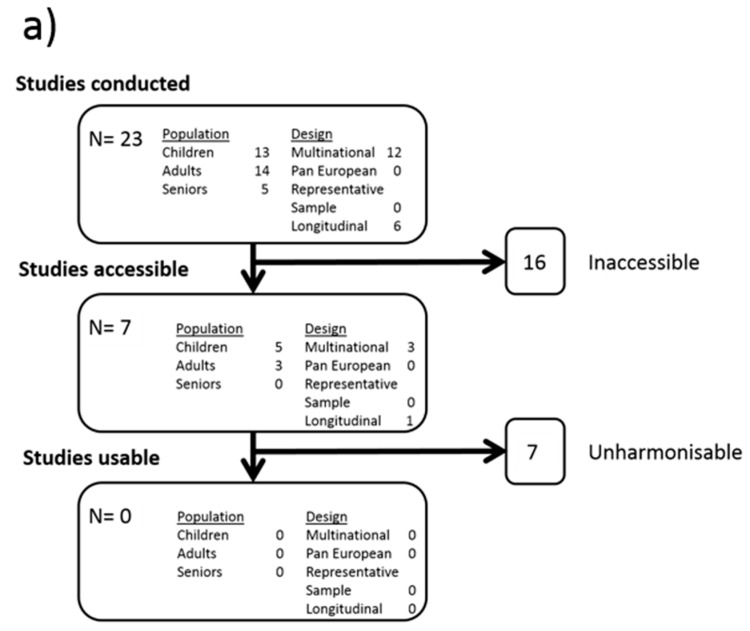

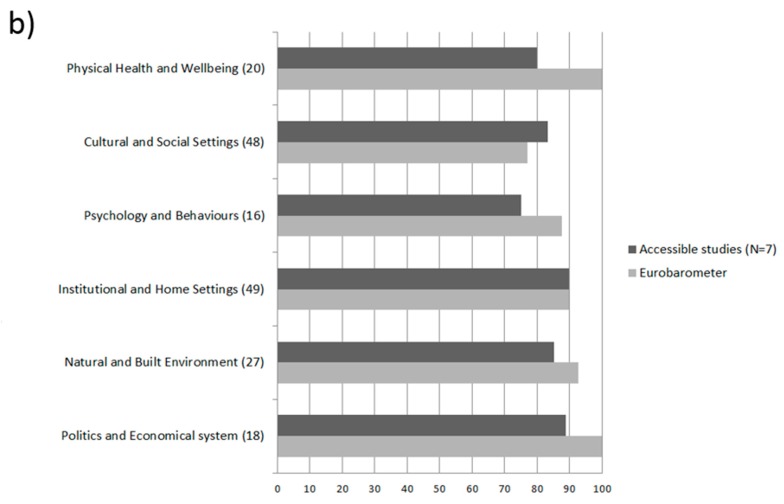
(**a**) Results of DEDIPAC inventory and data harmonisation of European datasets relevant to the determinants of sedentary behavior; (**b**) Proportion of determinants measured in the studies identified and Eurobarometer compared to those identified in the SOS framework that need to be investigated (presented by cluster of the SOS framework).

In addition, DEDIPAC has conducted an extensive inventory of existing datasets in Europe with data on determinants of sedentary behaviour identified through CORDIS, the European Commission’s primary public repository and portal, and through the DEDIPAC network. The aim was to harmonise and pool data for secondary analyses that would benefit from large sample sizes and the high natural variation found in Europe. The results of the inventory are shown in Figure 1a. Twenty three studies were identified, none were cross-European or representative of the diversity found in Europe although half were multi-country studies. However, gaining access to these datasets proved difficult due to various issues (e.g., relating to ethics, copyright and/or inability of the data custodian to share data). As such, only seven studies were accessible, and 16 studies ended up being inaccessible due to these data custody problems and the impossibility of sharing data because of data rights. Finally, the data from these studies were often too heterogeneous to be pooled. Data on determinants—and especially the more distal determinants—contained in these seven datasets covered only a very small proportion (Figure 1b) of the SOS framework. Also, data covered mostly the same personal factors as the studies included in the systematic reviews mentioned above and offered little prospect for new insights. Finally, there is a total dearth of exploitable longitudinal data which limits our ability to make causal inference.

Ongoing work within DEDIPAC suggests that by pooling surveillance data from the Eurobarometer (i.e., biannual, cross-sectional surveys conducted on behalf of the European Commission) and Eurostat (the directorate-general of the European Commission, responsible for statistical information of the European Union and its member states e.g., about economy and finance, industry, environment and energy), significant progress can be made in understanding the complex interactions between elements of the SOS framework and exploring the effect of distal (upstream) determinants such as macroeconomic factors and urbanisation. However, these proved to be the only exploitable sources of cross-European data, and as shown in Figure 1b they cover only a very small proportion of the determinants of sedentary behaviour.

## 5. Tackling the Sedentary Epidemic

The DEDIPAC consortium managed to organise and mobilise a tremendous research capacity which led to significant theoretical and knowledge progress about determinants of sedentary behaviour. Scarcity of high quality, EU-wide data collected with standardized methodology on both sedentary behaviours as well as a wide range of potential determinants, is a major barrier to this momentum and prevents progress towards informing policy and interventions to tackle the sedentary epidemic. Indeed, how can we base the design and implementation of interventions and policies on the partial and incomplete data that we have now? As current data are too limited to use Europe as a living laboratory, there is a clear need to collect new and robust data about the six clusters of determinants using a harmonised methodology across Europe, which is compliant with FAIR (Findable, Available, Interoperable, Reusable) principles [10].

## 6. Conclusions

In conclusion, we believe it is now time to take a broader view of the current research and policy in the field of sedentary behaviour, which is a widely-accepted threat to health. Data on determinants of engaging in sedentary behaviours are either non-existent, inaccessible or not usable for cross-European research. To progress to the development of effective policies and public health interventions to tackle the sedentary epidemic amongst European citizens, it is time to set up a pan-European cohort study on sedentary behaviour and its determinants. Ideally, this new cohort study should be longitudinal with repeated measures over a long follow-up period to include major life transition periods (such as retirement), cover a representative sample of European diversity over the life course and include vulnerable groups, and use up-to-date quantitative methodology (connected devices) as well as robust and sustainable self-reporting tools [11] to assess the variety of sedentary behaviours of interest as well as their potential determinants. Most importantly, these data should be FAIR-compliant and open to access to maximise utilisation and transdisciplinary research. Such data would enable identification of those determinants that are levers for change, in-silico simulation to forecast the impact and economic cost of potential interventions before they are implemented and monitor changes over time.

We should not sit on what we have (or have not). We should stand up to collect what is needed to tackle a central public health issue of the 21st century lifestyle.

## References

[1] Biswas A., Oh P.I., Faulkner G.E., Bajaj R.R., Silver M.A., Mitchell M.S., Alter D.A. (2015). Sedentary time and its association with risk for disease incidence, mortality, and hospitalization in adults: A systematic review and meta-analysis. Ann. Intern. Med..

[2] Ekelund U., Steene-Johannessen J., Brown W.J. (2016). Does physical activity attenuate, or even eliminate, the detrimental association of sitting time with mortality? A harmonised meta-analysis of data from more than 1 million men and women. Lancet.

[3] Ng S.W., Popkin B.M. (2012). Time use and physical activity: A shift away from movement across the globe. Obes. Rev..

[4] Ding D., Lawson K.D., Kolbe-Alexander T.L., Finkelstein E.A., Katzmarzyk P.T., van Mechelen W., Pratt M., Executive P.A.S. (2016). The economic burden of physical inactivity: A global analysis of major non-communicable diseases. Lancet.

[5] Lakerveld J., van der Ploeg H.P., Kroeze W., Ahrens W., Allais O., Andersen L.F., Cardon G., Capranica L., Chastin S., Donnelly A. (2014). Towards the integration and development of a cross-european research network and infrastructure: The Determinants of Diet and Physical Activity (DEDIPAC) knowledge hub. Int. J. Behav. Nutr. Phys. Act..

[6] Stierlin A.S., De Lepeleere S., Cardon G., Dargent-Molina P., Hoffmann B., Murphy M.H., Kennedy A., O’Donoghue G., Chastin S.F., De Craemer M. (2015). A systematic review of determinants of sedentary behaviour in youth: A DEDIPAC-study. Int. J. Behav. Nutr. Phys. Act..

[7] O’Donoghue G., Perchoux C., Mensah K., Lakerveld J., van der Ploeg H., Bernaards C., Chastin S.F.M., Simon C., O’Gorman D., Nazare J.A. (2016). A systematic review of correlates of sedentary behaviour in adults aged 18–65 years: A socio-ecological approach. BMC Public Health.

[8] Chastin S.F.M., Buck C., Freiberger E., Murphy M., Brug J., Cardon G., O’Donoghue G., Pigeot I., Oppert J.M., Consortium D. (2015). Systematic literature review of determinants of sedentary behaviour in older adults: A DEDIPAC study. Int. J. Behav. Nutr. Phys. Act..

[9] Chastin S.F., De Craemer M., Lien N., Bernaards C., Buck C., Oppert J.M., Nazare J.A., Lakerveld J., O’Donoghue G., Holdsworth M. (2016). The SOS-framework (systems of sedentary behaviours): An international transdisciplinary consensus framework for the study of determinants, research priorities and policy on sedentary behaviour across the life course: A DEDIPAC-study. Int. J. Behav. Nutr. Phys. Act..

[10] Wilkinson M.D., Dumontier M., Aalbersberg I.J., Appleton G., Axton M., Baak A., Blomberg N., Boiten J.W., Santos L.B.D., Bourne P.E. (2016). Comment: The fair guiding principles for scientific data management and stewardship. Sci. Data.

[11] Chastin S.F.M., Dontje M.L., Skelton D.A., Cukic I., Shaw R.J., Gill J.M.R., Greig C.A., Gale C.R., Deary I.J., Der G. (2018). Systematic comparative validation of self-report measures of sedentary time against an objective measure of postural sitting (activPAL). Int. J. Behav. Nutr. Phys. Act..

